# Comment on “Differential prognostic impact of liver resection by the site of concurrent extrahepatic diseases in patients with colorectal cancer liver metastases”

**DOI:** 10.1097/JS9.0000000000002974

**Published:** 2025-07-07

**Authors:** Xin-Chao Yang, Ya-Hui Sun, Xiong-Hui He

**Affiliations:** a Department of Surgery, Hainan Affiliated Hospital of Hainan Medical University; b Department of Gastrointestinal surgery, Hainan Affiliated Hospital of Hainan Medical University, Haikou, China

*Dear Editor,*

We read with great interest the article titled “Differential Prognostic Impact of Liver Resection by the Site of Concurrent Extrahepatic Diseases in Patients with Colorectal Cancer Liver Metastases^[1]^.” This study makes a significant contribution to understanding liver resection in patients with colorectal liver metastases (CRLM) and concurrent extrahepatic disease (EHD). However, I would like to bring to your attention a few critical issues that I believe need to be addressed in order to ensure the validity and generalizability of the study’s findings. This manuscript was prepared in accordance with the TITAN Guidelines 2025 for governing declaration and use of artificial intelligence in scholarly publishing^[2]^. No AI tools were used in the conception, design, analysis, interpretation, or drafting of this work.

Firstly, while the authors acknowledge the absence of RAS/BRAF mutation data in the limitations section, it is important to highlight that MSI (microsatellite instability) status, another critical biomarker with established prognostic relevance in colorectal cancer^[3,4]^, was not addressed in the study. Given the current significance of MSI-H (high microsatellite instability) in predicting favorable responses to immunotherapy^[5]^, its omission from the analysis represents a notable gap. The lack of MSI data limits our understanding of the tumor biology in this patient population, especially considering the heterogeneous nature of CRLM and its complex interaction with liver resection. Including MSI status would have provided more insight into the potential for immunotherapy and its impact on prognosis, further enhancing the study’s clinical applicability.

Secondly, although the study focuses on patients with CRLM and concurrent EHD, the absence of a clear definition or data on the control status of EHD, specifically whether it was resectable or responsive to other treatments, constitutes a significant limitation. The authors state that liver resection is indicated when EHD is controlled, but this key factor was not consistently measured or reported. Without this information, it is difficult to ascertain whether the observed survival benefits from liver resection are due to the surgery itself or to the better-controlled EHD in these patients.

Thirdly, while the authors correctly used inverse probability of treatment weighting (IPTW) to adjust for baseline differences between the hepatectomy and non-hepatectomy groups, it remains unclear whether this method fully addresses potential residual selection bias. Significant differences in clinical characteristics, such as tumor burden, prior treatment regimens, and the number of EHD sites, exist between the groups. Although IPTW adjusts for many of these factors, the potential for residual bias should be carefully considered when interpreting the overall survival benefits attributed to liver resection.

In conclusion, while the study presents valuable insights into the prognostic impact of liver resection in CRLM with concurrent EHD, these limitations highlight important areas that require further attention. I believe acknowledging and addressing these issues in future research will strengthen the conclusions and provide more robust evidence for clinical practice.

This manuscript was prepared in accordance with the TITAN Guidelines 2025 for governing declaration and use of artificial intelligence in scholarly publishing^5^. No AI tools were used in the conception, design, analysis, interpretation, or drafting of this work.

## Data Availability

The letter does not have any data.

## Research registration unique identifying number (UIN)

Not registered for the type of the study.
